# A Contribution to Medical Jurisprudence

**Published:** 1877-06

**Authors:** G. W. H. Kemper


### Selections.

A Contribution to Medical Jurisprudence.—*By G. W. H. Kemper, M.D.*—In December last, at the city of Muncie, Dr. J. P. McIlwain, of Eaton, Delaware county, Ind., was tried for the murder of Miss Mary Alice Foorman, a resident of the same locality. On account of the medical points involved, I have thought a synopsis of the case worth reporting. I shall aim, in all my statements, to be governed by the testimony of sworn witnesses.

On the 8th day of November, 1875, Miss Foorman, aged nineteen years, and supposed to be pregnant, mysteriously disappeared from her home, and all attempts to fathom the mystery were fruitless until the last of April, 1876, when certain members of her family made disclosures that led to the arrest of Drs. J. P. McIlwain and H. B. Manzer; thus, too, it was learned that the girl was dead, and buried near Eaton. The authorities at once offered a reward for her body, and on Sunday, May 7, 1876, at four o'clock P.M., the body was discovered and exhumed.

The body was buried in a wet portion of ground; the grave was about two feet deep, dug in an excavation caused by a tree having been blown over by its roots. About six inches of soil and some pieces of wood were thrown over the body, and all under water of some depth. The soil was so loose that the finder dug out the corpse simply with his hands. The body was dressed in a pair of cotton hose, cotton drawers, chemise, and night-gown. A woolen shawl lay across the chest, and the body was wrapped in a coarse woolen blanket.

Immediately after exhumation, the coroner of that county (Blackford) was notified that an inquisition was held. Drs. W. C. Ransom and P. Drayer, of Hartford, made a post mortem examination. The features were well preserved, and looked quite natural for several hours after disinterment. A large number of witnesses recognized the body as that of Mary A. Foorman.*

I saw the body the following morning at half past seven o'clock, at the village of Eaton, where it had been removed for burial. It was thought that the examination of the previous day was not sufficiently complete, and accordingly Drs. Ransom and Drayer were notified by telegraph, and arrived at noon, and proceeded at once with a further examination. Drs. James, Boyden, Leech, and myself, all of Muncie, witnessed the dissection. I made the following notes:

*Second Examination—Twenty Hours after Exhumation.*—Odor very offensive. All the hair was absent from the scalp, except a small patch at occiput. The cuticle was detached at various places of the body, of the extremities, and also of the mammary glands, the areolæ being thus destroyed. The pubic hairs had fallen off. There was no blood present in the blood vessels, nor in any cavity of the body. Section by Dr. W. C. Ransom.

The *brain* was first exposed by removing the calvarium. The dura mater was intact, and presented an ashen gray color. The whole mass appeared shrunken, so that the cranium was not completely filled. The brain substance was disorganized, and spread out when the dura mater was removed. Odor not so offensive as is usual after decomposition begins.

The *lungs* were *in situ*—collapsed. They presented a dark purplish hue, and were in a very good state of preservation.

The *heart* had been removed at the previous dissection, examined and replaced in the thoracic cavity. Its interior was exposed, and all the appearances indicated a normal condition. The organ was well preserved.

The *liver* was a decomposed and putrid mass.

The *uterus* had been removed the previous evening, placed in a glass jar, and sealed. It was exhibited at this time for

*The fact of this recognition is remarkable. She was buried one hundred and seventy-eight days—twenty-five and a half weeks. "On a trial that took place some years since at Edinburgh, for stealing subjects, where the body had been interred nine weeks before the recognition, Dr. Barclay, the anatomist, testified that the longest time he ever knew during which the features remained recognizable, was a fortnight. Yet a witness swore particularly to the identity of the body." (Beck's Med. Jurisprudence, Vol. II., 12th ed., page 49.)

inspection. It was enlarged, weighing seven and a half ounces avoirdupois. It measured from os to fundus seven and a half inches, and the greatest width four and a half inches. The walls were half an inch in thickness and spongy. The entire organ was well preserved. The internal surface of the body was denuded of mucous membrane, and presented a dark red color. A spot situated near the fundus indicated a placental site. The os was patulous, its diameter being about three-fourths of an inch.

The *stomach* had been removed and preserved in a glass jar.

The testimony of Dr. McIlwain, the defendant, was in substance as follows: Miss Foorman called to consult me at three different times in the latter part of October, 1875, in regard to the cessation of her menses. She admitted the possibility of pregnancy. I refused to give her medicine; and at the last interview she requested me to visit her mother, and have a conversation with her on the subject, which I did. Her mother supposed her to be three or four months advanced in pregnancy; and stated that Nathan Smith and her daughter were engaged, but she preferred that she should not marry while in that condition. I refused to furnish any means for procuring an abortion, and recommended that she be taken to the Home of the Friendless at Fort Wayne, where she could be cared for during her pregnancy and confinement. I agreed to take her to Fort Wayne. I also had a conversation with Mrs. Foorman's son John, about the arrangement, and he and I went to her residence on the evening of November 8th, and brought Mary in a buggy to my house, where we arrived about ten o'clock. Being hindered by business, I could not leave, and she remained at my house for three days. On Thursday, November 11th, at ten o'clock A.M., Mary had a chill, and a second at four o'clock P.M. I made no particulrr examination, as I supposed it to be ague. She complained of feeling cold, and I placed an additional quilt upon her. I left the room and returned in about half an hour; she still complained of feeling cold, and asked for more covering. I went in again in a few minutes and found her shaking; muscles rigid. I then went and brought Dr. Manzer. I made no close examination, but found her pulse rapid. She was in spasms, hands clenched and arms rigid. Did not examine pupils. She complained of nothing but cold, and called frequently for water. She continued in about that state until she died. She was conscious until a few minutes before she died. After death blood issued from her mouth and nose. There was no hemorrhage from her at any time while in my house, except from mouth and nose, and no stains in the bed where she lay.

Dr. Manzer testified in substance as follows: Felt her pulse, and found it from 100 to 130. Her face was flushed, eyes more or less protruded, and pupils dilated. I asked Dr. McIlwain what he had been doing, and he said nothing. She had a number of spasms after I arrived. I asked her what she had been taking, and she said oil of tansy and oil of savin. After death her features were natural. After we had buried her and returned to Dr. McIlwain's house, I saw a broken goblet in the room which the deceased had occupied. There was something in it; did not know what it was—a dark fluid, with a small amount of white precipitate at the bottom; it was bitter to the taste and odorless. I believe she died from strychnia poisoning. Was not aware that convulsions attend death from hemorrhage.

The girl died at six o'clock P.M., eight hours after the first chill. During all this time they state no effort was made to administer an antidote, or in any way alleviate her sufferings.

Dr. McIlwain further stated: I never attempted an abortion upon Mary by any means, and do not know from my own personal knowledge that she was pregnant.

The body, the night she died, was secretly buried by the two physicians and Robert Brandt.

With this general history, we are now prepared to consider the medical evidence bearing upon the case, and will first take up the question, *was Mary A. Foorman pregnant?* Nearly all the medical witnesses concurred in the opinion that she was. The principal reasons for thinking so were as follows:

First. The appearances of the uterus. It was enlarged, being seven and a half inches long and four and a half inches wide, and weighed seven and a half ounces avoirdupois. Taken in connection with other symptoms, the physicians for the State, namely, Drs. W. C. Ransom, of Hartford; T. B. Harvey, W. B. Fletcher, and W. M. Bullard, of Indianapolis; G. D. Leech and G. W. H. Kemper, of Muncie, considered this condition produced by pregnancy. The physicians for the defense, namely, Drs. H. C. Winans and T. J. Bowls, of Muncie, denied the pregnancy, and attributed the enlargement of the uterus to post mortem changes, or probably to hæmatometra. Dr. Winans thought the enlargement was caused by stretching, due to the generation of gas within its cavity caused by the process of decomposition. That the hypothesis of post mortem changes was untenable, is apparent when it is remembered that the entire body was in a comparatively good state of preservation, and the uterus is well known to be the last organ of the body to decay.* Again, there was no good reason for supposing a hæmatometra. Acquired imperforation of the vulvo-uterine canal is rare, even in multipara. Her mother stated that menstruation began at the age of fifteen, and continued regularly until August preceding her death, so that from that time until her death she could not have passed over more than three or four menstrual periods; and furthermore, sexual intercourse was acknowledged by her lover.

Second. A well defined spot, situated on the left side of the fundus, at which point the uterine wall *was thinner*, and opening into which were four or five sinuses that were readily inflated with a blow-pipe by Dr. Fletcher, was called by the physicians for the State a placental site. Drs. Winans and Bowls denied and pronounced it a "decayed spot." Both of these gentlemen affirmed that the uterine wall was *thicker* instead of thinner at the placental site. Dr. Bowls said that he would expect to find the walls of the uterus, at the fifth month, one to one and a half inches thick, and the placental site very much the thickest. As this is a point of some importance in medico-legal cases, and but little assistance rendered by the books, I would call attention to a recent examination by Dr. Fletcher, where it is conclusively shown that the placental site is *thinner*.†

---

* "It is worthy of remark that the uterus resists decomposition more than other internal organs. In a case in which the body of a female, who had been missing nine months, was found and examined, although all other parts were completely decomposed, the uterus was of a reddish color, firm in structure, and its parts were recognizable, so that Casper, who examined the case, was able to affirm that the female was not pregnant at the time of her death." (Taylor's Med. Jur., 5th Am. ed., p. 545.)

† American Practitioner, April, 1877, p. 226.

Third. Another point of importance was involved, namely, the value of the microscope in determining, so long a period after death, whether the woman had been recently delivered. Dr. Flether, by this instrument, found the walls of the uterus denuded of mucous membrane, and considered that this was a proof that the woman had been recently delivered. He considered this evidence strengthened from the fact that he found the mucous membrane of the cervix present, and in a good state of preservation, and that the mucous membrane of the mouth and intestines was well preserved. Dr. Winans examined with a lens of sufficient "power to exhibit the fiber in white writing paper," and affirmed that he found the mucous membrane of the body of the uterus intact, except at one point in the upper part and to the left, which he designated the "decayed spot." He admitted that delivery at the fifth month is always indicated by the shedding of the mucous coating. Dr. Bowls also thought the mucous membrane was present even at this time (December, 1876), and yet in a short time he declared that the uterus was so much decayed that he was in doubt whether the enlargement was due to the retention of blood or not. Likewise, Dr. Winans stated, on the cross examination, that the uterus was in an advanced stage of decomposition. Query: If there was any putrefactive changes at all of the uterus, would not the mucous membrane yield first?

Fourth. The dilatation of the os—about three-fourths of an inch—was regarded by the physicians for the State as evidence of recent delivery; those for the defense asserting that it resulted from hæmatometra.

Fifth. Another point at issue was in regard to the structure of the uterus. Drs. Winans and Bowls affirmed that there was no muscular tissue in the unimpregnated uterus, that it is "simply a connective tissue" (Winans); "a potential fiber" (Bowls). According to Dr. Winans, no fibers were visible in this uterus; *ergo*, it was unimpregnated. Further on in his testimony, when describing the depth of the "decayed spot," he forsook his theory and said: "Its deepest portion was in a plane with the outermost layer of the muscular tissue." Question by Mr. Ryan: "Through the muscular coats? Answer: "Yes, sir; and partially the third." Dr. Fletcher showed the difference, which was merely one of development, between the muscular fibers of the unimpregnated and of the impregnated uterus; and demonstrated that the fibers of this uterus corresponded in size and appearance with the latter.

The next question involved is the *cause of death of Mary A. Foorman.* Upon this question also the physicians for the State and those for the defense failed to agree. We will first consider poisoning as a cause. There is no evidence to this effect, except the statements of Drs. McIlwain and Manzer. Drs. Winans and Bowls, basing their conclusions upon this testimony, gave an opinion that she died from strychnia poisoning. On the contrary, Dr. W. M. Bullard, who analyzed the stomach, testified that the organ was empty, healthy, and well preserved. The proper tests known to chemists were applied for strychnia as well as the other usual poisons. No trace of any poison was detected, and he gave an opinion that none was present. He cited cases where arsenic and strychnia had been detected in the dead body after a much longer period than had elapsed in this case, thus proving that poison would have been detected had it produced death.

We next proceed to notice the uterine lesions which might point to a cause of death. Dr. Harvey, who examined the uterus on the 6th day of September, 1876, testified that it was well preserved and healthy, except that the mucous membrane of the body was absent, and that the neck or mouth had been considerably lacerated, indicating instrumental violence. He thought the uterus had been impregnated and the fœtus had been expelled by mechanical means. He thought hemorrhage was the immediate cause of death. In the main, the physicians for the State concurred in these opinions.

I have not deemed it proper to add the testimony of non-professional persons, as it was the medical side of the question I desired to consider. I will state, however, that evidence was adduced confirmatory of pregnancy, as well as the production of abortion. The very manner of disposing of the body is stamped with guilt, and the lashing of a guilty conscience in John Foorman, brother of the deceased, who was cognizant of all the facts, would not let him rest, and he eventually confessed all, adding additional proof to the maxim that "murder will out."

The jury returned a verdict of guilty, and sentenced Dr. McIlwain to the State prison for two years.—*Am. Practitioner.*

